# Chinese cases of early infantile epileptic encephalopathy: a novel mutation in the PCDH19 gene was proved in a mosaic male- case report

**DOI:** 10.1186/s12881-018-0621-x

**Published:** 2018-06-04

**Authors:** Yuxia Tan, Mei Hou, Shaochun Ma, Peipei Liu, Shungang Xia, Yu Wang, Liping Chen, Zongbo Chen

**Affiliations:** 1 Department of Pediatrics, The Affiliated Hospital of Qingdao University, No. 59, Haier Road, Qingdao, 266000 China; 2 Department of Pediatrics, Zibo City Maternal and Child Health Hospital, Zibo City, 255029 Shandong Province China; 3 Department of Pediatric Rehabilitation, The Affiliated Qingdao Women & Children’s Hospital of Qingdao University, Qingdao, 266034 China; 4 Department of Pediatric Neurology, The Affiliated Qingdao Women & Children’s Hospital of Qingdao University, Qingdao, 266034 China

**Keywords:** *PCDH19*, Infantile epilepsy, Clinical manifestations, Gene variations

## Abstract

**Background:**

The link between the protocadherin-19 (*PCDH19*) gene and epilepsy suggests that an unusual form of X-linked inheritance affects females but is transmitted through asymptomatic males. Individuals with epilepsy associated with mutations in the *PCDH19* gene display generalized or focal seizures with or without fever sensitivity. The clinical manifestation of the condition ranges from mild to severe, resulting in intellectual disability and behavioural disturbance. In the present study, we assessed mutations in the *PCDH19* gene and the clinical features of a group of Chinese patients with early infantile epileptic encephalopathy and aimed to provide further insight into the understanding of epilepsy and mental retardation limited to females (EFMR; MIM 300088).

**Case Presentation:**

We described three variations in the *PCDH19* gene in Chinese patients with epilepsy who developed generalized seizures occurring in clusters with or without triggering by fever. Candidate genes were screened for mutations that cause epilepsy and related paroxysmal or nervous system diseases in the coding exons and intron–exon boundaries using polymerase chain reaction (PCR) of genomic deoxyribonucleic acid (DNA) followed by sequencing. The variations were sequenced using next-generation sequencing technology and verified with first-generation sequencing. Exome sequencing of a multigene epilepsy panel revealed three mutations in the *PCDH19* gene in a mosaic male and two unrelated females. These included a frameshift mutation c.1508_1509insT (p.Thr504HisfsTer19), a missense mutation c.1681C > T (p.Pro561Ser) and a nonsense mutation c.918C > G (p.Tyr306Ter). Of the three mutations in the *PCDH19* gene associated with early infantile epileptic encephalopathy, the frameshift variation in a mosaic male is novel and de novo, the missense variation is de novo and is the second ever reported in females, and the nonsense variation was inherited from the paternal line and is the first example discovered in a female.

**Conclusions:**

The results from our current study provide new insight into and perspectives for the molecular genetic link between epilepsy and *PCDH19* alterations. Moreover, our new findings of the male mosaic variant broaden the spectrum of *PCDH19*-related epilepsy and provide a new understanding of this complex genetic disorder.

**Electronic supplementary material:**

The online version of this article (10.1186/s12881-018-0621-x) contains supplementary material, which is available to authorized users.

## Background

Epilepsy and mental retardation limited to females (EFMR; MIM 300088), first reported by Juberg et al. (1971), affects females but is transmitted through unaffected males, following an unusual X-linked form of inheritance [1]. EFMR, also known as early infantile epileptic encephalopathy-9 (EIEE9, OMIM# 300088), is a genetic disorder, of which the de novo or familial heterozygous variation of the *PCDH19* gene (OMIM 300460) is forecasted to be the second most common cause in addition to variations of the sodium voltage-gated channel alpha subunit 1 (*SCN1A*) gene [2–5]. Since the initial description of the *PCDH19* gene, the evidence of a link between the clinical features of epilepsy and de novo or familial mutations in the *PCDH19* gene has increased. These links include infantile onset of multiple seizure types, such as Dravet syndrome, generalized or focal epilepsy with or without fever sensitivity, and epilepsy of variable severity in females with or without intellectual disability, behavioural disturbance or psychiatric symptoms [2, 6–8].

The *PCDH19* gene is located on the X chromosome (Xq22.1) and encodes protocadherin-19. This is a calcium-dependent adhesion molecule belonging to the δ2-protocadherin (pcdhs) subclass of the cadherin (cdhs) superfamily [9]. The *PCDH19* gene shows an unusual X-linked form of inheritance affecting only females but being transmitted via asymptomatic males, which implies that it is pathogenic in heterozygous mutated females but non-pathogenic in hemizygous mutated males [6, 10]. *PCDH19* has been shown to be expressed in the central nervous system and is possibly involved in a cellular interference mechanism that affects brain development, neuronal connections, and intracellular signal transductions at the synaptic membrane [3, 11, 12].

Variations in the *PCDH19* gene were first reported by Dibbens et al. (2008) in several families with the EFMR disorder [10]. Depienne et al. (2009) discovered that subjects with *PCDH19*-related disease might be normal during early development but experience seizure onset occurring in clusters with fever sensitivity, an observation similarly seen with Dravet syndrome (DS) [6]. An increasing number of female epilepsy patients with *PCDH19* mutations have been reported over recent years, and the clinical spectrum of diseases associated with *PCDH19* gene mutations has also expanded, including patients displaying focal or generalized seizures recurring in clusters or isolation with or without febrile trigger, ranging from mild to severe, with or without cognitive impairment, behavioural disturbance, and psychiatric symptoms [2, 4, 11, 13].

Up to now, the molecular genetic study for *PCDH19*-related epilepsy has been focused on females. Male subjects were generally excluded because of the unusual X-linked inheritance and the pathogenic hypothesis associated with the cellular interference mechanism. However, following the report of a male with a mosaic *PCDH19* deletion [6], a small number of recent studies have described several alterations of the *PCDH19* gene in mosaic male patients in succession [14–17].

Here, we present evidence of three mutations of the *PCDH19* gene in a mosaic male and two unrelated females in Chinese individuals with epilepsy, further contributing to the clinical understanding of EFMR in relation to the *PCDH19* gene.

## Case presentation

We recruited three Chinese children with epilepsy treated in the Qingdao Women & Children’s Hospital between March 2016 and April 2017. The clinical diagnosis was made by clinical geneticists. Additional clinical information relevant to the diagnosis was provided by the parents and the caregivers. The CARE guidelines were followed in reporting these cases. Two millilitres of venous blood was collected from each patient and each parent of the subject upon signed informed consent. Then, the blood samples were sent to Beijing Kangso Medical Inspection for gene testing: nervous system diseases “big bag” for patient 1 (a total of 1906 genes) and epilepsy and related paroxysmal diseases for patient 2 and patient 3 (a total of 518 genes). Genomic DNA from blood samples was extracted using the Qiagen FlexiGene DNA kit (Qiagen, Germany) following the manufacture’s guidelines. Candidate genes were screened for mutations in the coding exons and intron–exon boundaries using PCR of genomic DNA followed by sequencing. Primer sequences and PCR conditions are available upon request. The variations were sequenced with next-generation sequencing and verified with first-generation sequencing. Detailed experimental methods can be found in Additional file 1.

### Patient 1

Patient 1 is a 27-month-old male. He was born via normal vaginal delivery at full-term. He weighed 3.0 kg at birth. No neurological problems were noted during routine examinations at birth. The pregnancy and perinatal history were unremarkable. He was the first child of non-consanguineous parents. Family history was noncontributory. At the age of 7 months, his first recurrent, afebrile generalized tonic seizures (TS) were noted. The video electroencephalogram (VEEG) showed sharp, spike waves with partial origin at right frontal regions and high voltage slow background. The brain magnetic resonance imaging (MRI) was normal. Following treatment with valproate (VPA) and topiramate (TPM) for 5 months, his cluster seizures remained and occurred from time to time. After adjusting the antiepileptic drugs (AED) to VPA and phenobarbital (PB), the control of seizures remained unsatisfactory. After 9 months from the initial onset, oxcarbazepine (OXC) was added. Then, his seizures were gradually controlled with VPA, PB and OXC, and he remained seizure-free for 3 months till the last follow-up.

In his development, his early psychomotor skills were unremarkable. However, he showed signs of intellectual impairment and behavioural disturbance following the onset of his seizures. His head control was complete at 2 months. He rolled at 4 months, sat up unsupported at 7 months, climbed at 11 months, and walked independently at 13 months. At present, he runs unsteadily and falls easily when standing up from squatting. He spoke his first word at age 15 months and had only three purposeful words by 24 months. He was unable to act following orders or to express his wants accurately. He receives language training and physical therapy at present.

**Fig. 1:**
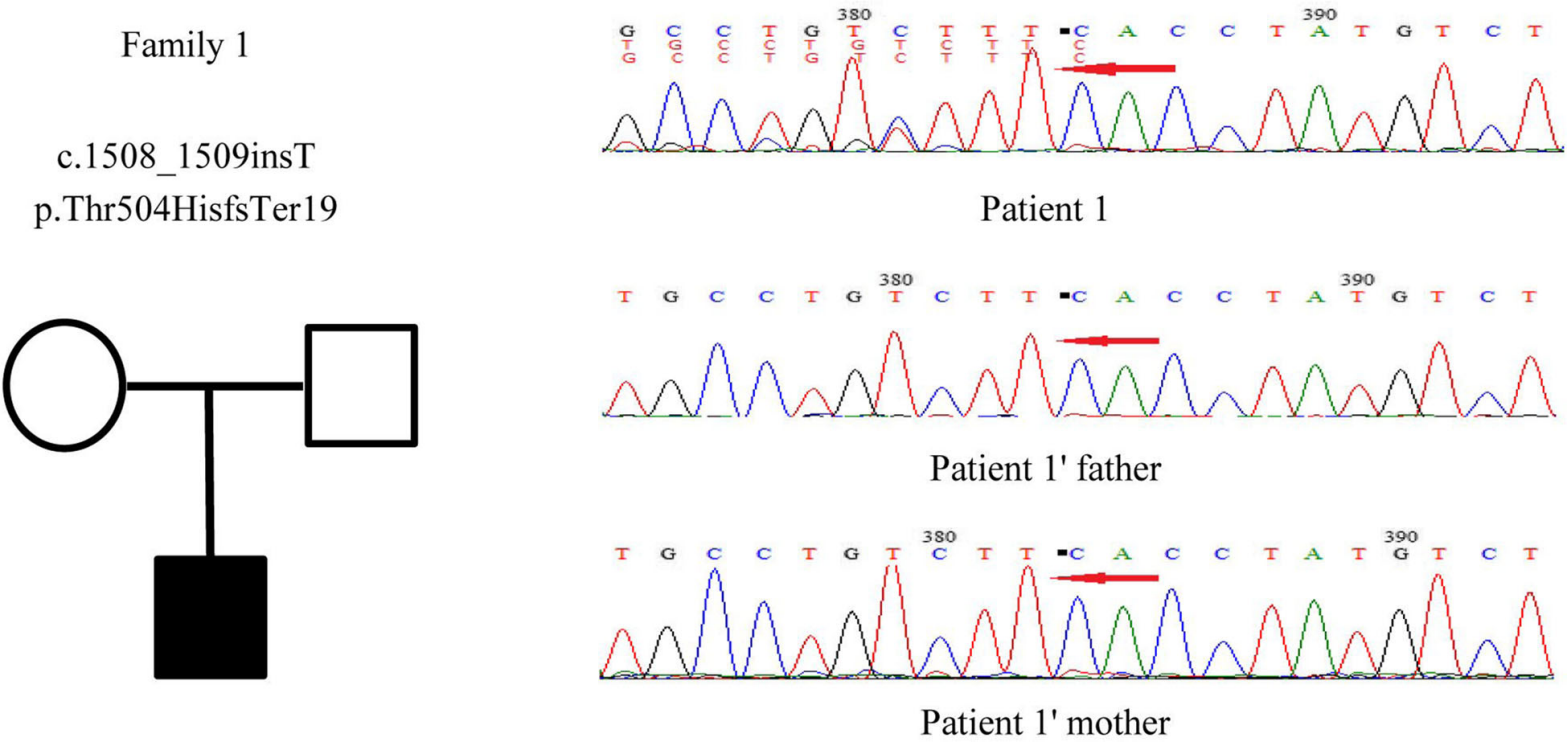
The genetic map and mutation sequence chromatograms in the *PCDH19* gene of family 1. Black square: affected mutation-carrying male; white square: male without *PCDH19* mutation; white circle: female without *PCDH19* mutation. The red arrows indicate the location of the identified mutation

By clinical exome sequencing, patient 1 was found to have a frameshift mutation in the *PCDH19* gene: a thymine (T) inserted into the encoding region between nucleotides 1508 and 1509 (c.1508_1509insT), suggesting a mosaic nucleotide mutation. The mutation led to the synthesis of amino acid beginning from amino acid 504 of Thr changed and ending in the nineteenth amino acid after the change (p.Thr504HisfsTer19). This mutation was a de novo variation. The male’s parents showed no abnormalities (Fig. 1). The Sanger sequencing results of both strands covering the variant on Integrate Genome Viewer are shown in more detail in Additional file 2.

### Patient 2

Patient 2 is a 34-month-old female. She was born at full-term via normal vaginal delivery without distress and dysmorphic features. At birth, she weighed 3.3 kg, and neurologic examination suggested no abnormalities. The perinatal history, pregnancy and neonatal course were all unremarkable. She is the only child to her healthy parents. She had no family history of neurological disease. Her first seizure displaying a tonic seizure (TS) was noted during a febrile illness at the age of 14 months; the EEG and brain computed tomography (CT) then were normal. By 19 months of age, she developed a cluster of febrile seizures lasting for approximately 10 s each time, with seizures occurring 6 times in 36 h. At the age of 31 months, a second cluster appeared, triggered by fever. The VEEG and brain MRI were also normal at this onset. Her psychomotor development was unremarkable. Her seizure was gradually controlled after being given TPM.

**Fig. 2:**
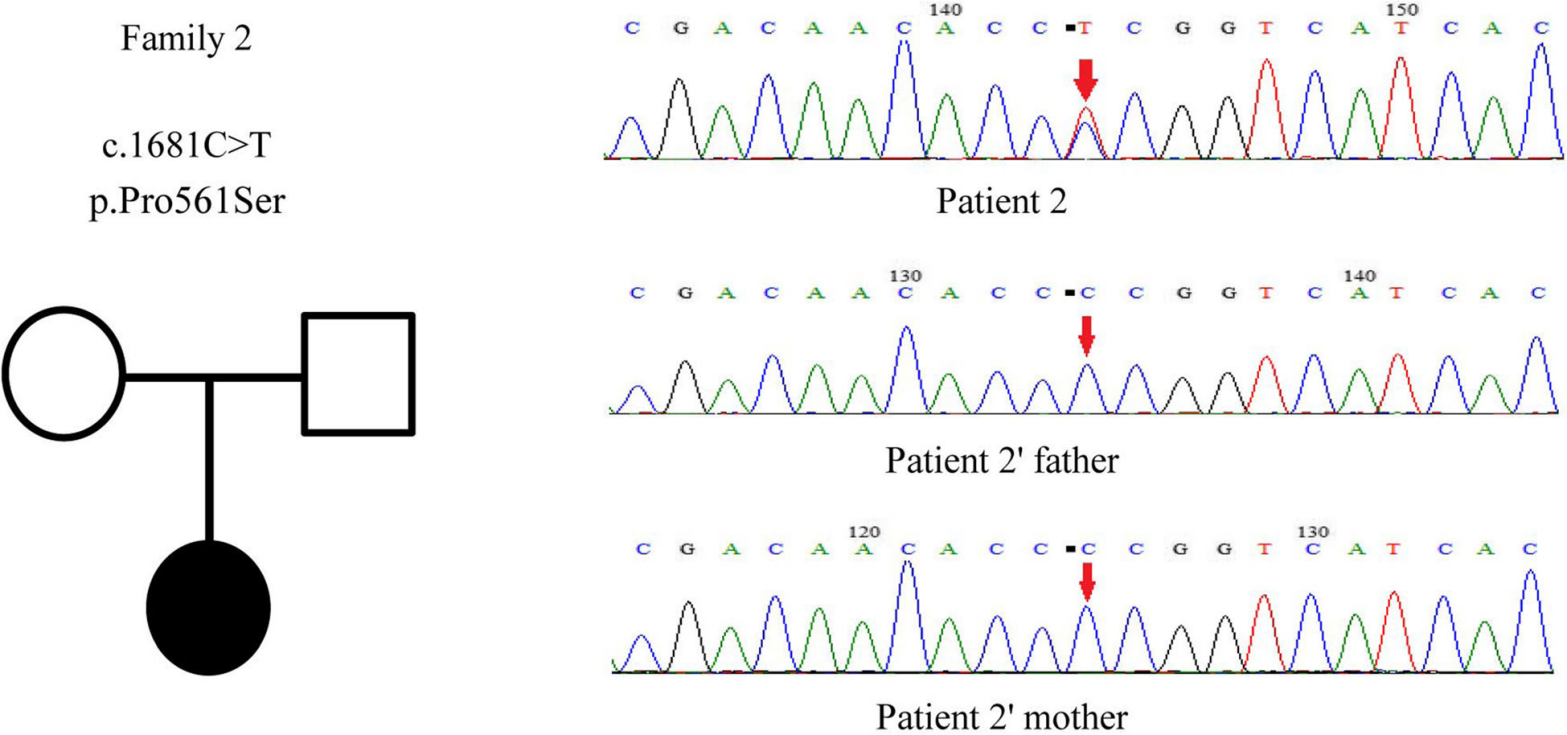
The genetic map and mutation sequence chromatograms in the *PCDH19* gene of family 2. Black circle: affected mutation-carrying female; white circle: female without *PCDH19* mutation; white square: male without *PCDH19* mutation. The red arrows indicate the location of the identified mutation

Clinical exome sequencing revealed a missense mutation in the *PCDH19* gene: a cytosine to thymine (C > T) nucleotide change at position 1681 (c.1681C > T) in patient 2, a heterozygous nucleotide mutation causing amino acid 561 to change from Pro to Ser (p.Pro561Ser). This mutation was de novo, and this alteration was not found in her parents (Fig. 2).

### Patient 3

Patient 3 is a 39-month-old female. She was born at full-term via a normal vaginal delivery. She weighed 3.18 kg at birth, with no apparent neurological abnormalities. The prenatal history and neonatal development were normal. She is the only child of non-consanguineous parents. Her grandmother has a history of seizures. Her seizure onset was at 20 months when she experienced a cluster of afebrile generalized tonic and clonic seizures (GTCS), with seizures occurring 6 times in a day. The VEEG and brain MRI showed no abnormality at that time. After a one-month seizure-free period, she presented with a second cluster of afebrile generalized seizures. The VEEG showed sharp slow wave emission mainly in the right occipital region. Treatment with levetiracetam resulted in gradual control of seizures. She requires no other significant medical attention.

**Fig. 3:**
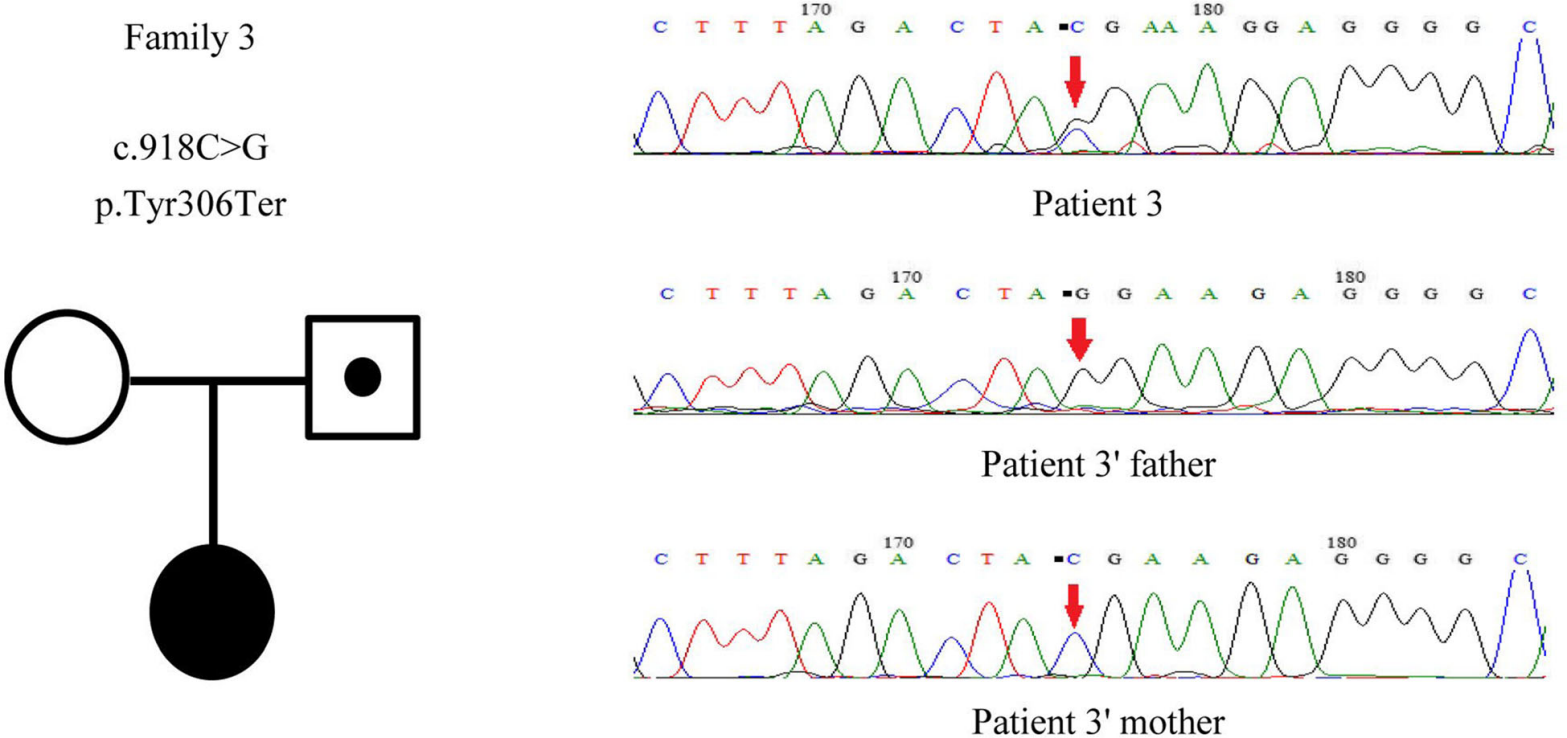
The genetic map and mutation sequence chromatograms in the *PCDH19* gene of family 3. Black circle: affected mutation-carrying female; white circle: female without *PCDH19* mutation; a dot in a white square: asymptomatic mutation-carrying male. The red arrows indicate the location of the identified mutation

For patient 3, clinical exome sequencing suggested a nonsense mutation in the PCDH19 gene: acytosine to guanine (C > G) nucleotide change at nucleotide 918 (c.918C > G), a heterozygous nucleotide mutation. The mutation changed amino acid 306 from Tyr into a termination codon (p.Tyr306Ter). The mutation was inherited from the paternal line. The girl’s father was identified to be a hemizygote, but her mother did not carry this alteration (Fig. 3).

## Discussion and Conclusions

We identified three mutations of *PCDH19*-related epilepsy in a cohort of Chinese children. The clinical data and epileptogenic characters of the three patients carrying *PCDH19* variations are described in Table 1.

**Table 1:** Clinical data of patients with epilepsy carrying *PCDH19* mutations

Patient number	Male patient 1	Female patient 2	Female patient 3	Male patient (Terracciano et al. 2016) [14]
PCDH19 mutation	c.1508_1509insT	c.1681C > T	c.918C > G	c.918C > G
	p.Thr504HisfsTer19	p.Pro561Ser	p.Tyr306Ter	p.Tyr306*
Mutation type	Frameshift mutation	Missense mutation	Nonsense mutation	Nonsense mutation
Sex	Male	Female	Female	Male
Present age	2 y 3 m	2 y 10 m	3 y 3 m	4 y
Birth weight	3.0 kg	3.3 kg	3.18 kg	Unknown
Age at onset	7 m	1 y 2 m	1 y 8 m	9 m
Type of seizures at onset	TS	TS	GTCS	Focal with SG
Cluster occurrence	Yes	Yes	Yes	Yes
Fever sensitivity	No	Yes	No	Yes
Status epilepticus	No	No	No	Yes
EEG	Abnormal	Normal	Abnormal	Normal
MRI	Normal	Normal	Normal	Normal
Intellectual disability	Yes	No	No	Borderline
Psychiatric symptoms	No	No	No	No
Behavioural disturbance	Yes	No	No	Yes
Current AED	Phenobarbital, valproate, oxcarbazepine	Topiramate	Levetiracetam	Valproic acid
Persistence of seizures with AED	Yes	No	No	No
Transmission	De novo	De novo	Paternal inheritance	De novo

In a 2016 study, Terracciano et al. revealed a male patient with the nonsense mutation c.918C > G (p.Tyr306Ter), the same position as patient 3 in our cohort. Here, we compared the findings from our study with those of Terracciano et al. [14]. Of the four patients, seizures started within the first year of life for two patients and after the first year of life for the other two, with an average age of onset of 12 months (6–20 months). All patients had generalized seizures or focal seizures with secondary generalization (SG), characterizing the onset of epilepsy. Cluster seizures occurred in all subjects, and patient 4 had confirmed epileptic status. Two patients had seizures associated with fever sensitivity at onset, and the other two were afebrile onset. EEG was normal in patients 2 and 4 but showed abnormality in patients 1 and 3. Brain MRI had no special findings. Psychiatric symptoms were absent from all the patients. However, patients 1 and 4 developed intellectual impairment and behavioural disturbance. Patient 1 had recurrent epilepsy despite being treated with multiple anti-epileptic drugs, suggesting that the seizures related to *PCDH19* variations were difficult to control to a certain degree.

Despite the many clinical manifestations of *PCDH19*-related epilepsy, the disease shares some common characteristics, namely, onset at the age of infancy and babyhood, generalized seizures and cluster seizures [3]. Generalized tonic seizures (TS), tonic-clonic seizures (GTCS) or focal seizures with SG occurring in clusters with or without fever sensitivity are the common seizure types. Other seizure types, such as myoclonic seizures, atonic seizures and atypical absences, are rare. Intellectual development abnormalities vary from normal to severe impairment. Patients may or may not display psychiatric symptoms and behavioural disturbance [4, 13].

Of the four patients, three genetic alterations were de novo, and one was inherited from her asymptomatic father. *PCDH19* mutations in most patients are sporadic and de novo, whilst some are of paternal inheritance. This makes it difficult to recognize the pattern of inheritance [18, 19]. To determine the complete feature of EFMR, more patients need to be studied.

With the progress of genetic detection technology, thus far, over 150 *PCDH19* mutations have been identified, featuring small deletion, small insertion, nonsense, missense, microduplication, intragenic deletion, splice site mutation and whole gene deletion. The great majority of the variations (over 90%) are located in exon 1, the largest exon encoding the extracellular cadherin domain [5]. In our study, we report three mutations located in exon 1, including a frameshift mutation, a missense mutation and a nonsense mutation. One mutation was novel, and two were reported recently. The frameshift alteration c.1508_1509insT (p.Thr504HisfsTer19) was identified in a mosaic male, which was a novel and de novo mutation. This discovery expands the spectrum of *PCDH19*-related epilepsy in males. A brief review of mosaic males in terms of the *PCDH19* gene are as follows: Stosser et al. (2017) identified five males with mosaic pathogenic variants presenting with epilepsy with or without signs of developmental delay, similar to female patients with *PCDH19* pathogenic variants [20]. Other researchers also reported several variants in mosaic males, and these male patients with mosaic mutations showed similar clinical features of epileptic encephalopathy to those presented in *PCDH19* affected females. The previously described pathogenic mutations in mosaic males contain frameshift mutation, nonsense variant, missense mutation of exon 1 and alteration located in the splice site of intron 1 [14, 16, 17, 20]. Nevertheless, here, we attest the first male with the *PCDH19* gene pathogenic variant c.1508_1509insT, which contributes to further explanation of this complex and an interesting genetic disorder.

The missense mutation c.1681C > T (p.Pro561Ser) was reported in a female patient by Carvill in 2013 [21] and the nonsense mutation c.918C > G (p.Tyr306Ter) in a mosaic male patient by Terracciano in 2016 [14]. These reports together with our findings collectively suggest that *PCDH19* gene mutations have a significant association with epilepsy. The missense alteration was de novo. The nonsense mutation was transmitted from an asymptomatic father, but the transmitting male showed no abnormalities because of the unusual X-linked inheritance of *PCDH19*.

**Fig. 4:**
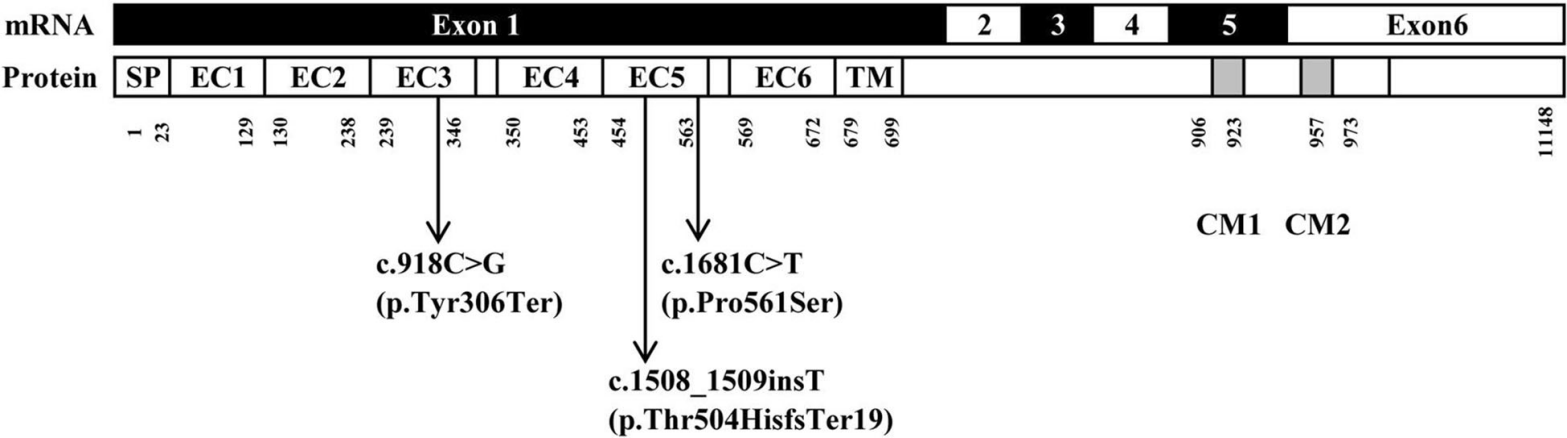
Schematic diagram of the point mutations identified in the *PCDH19* gene. SP: signal peptide; EC: extracellular cadherin domain; TM: transmembrane domain; CM1 and CM2; cytoplasmic domains 1 and 2

The full-length mRNA of the *PCDH19* gene is 9765 nucleotides, encoding a 1148 amino acid protein. The *PCDH19* gene consists of six exons; exon 1 is the largest exon encoding a signal peptide, the whole extracellular domain and a short transmembrane domain (TM), including six repeats of EC cadherin regulating cell-cell interactions [10]. All three mutations we identified in the present study were found in exon 1 of the *PCDH19* gene, with the nonsense mutation c.918C > G (p.Tyr306Ter) in EC3 and both the frameshift mutation c.1508_1509insT (p.Thr504HisfsTer19) and the missense mutation c.1681C > T (p.Pro561Ser) in EC5 (Fig. 4). The nonsense and missense mutations were predicted to be deleterious in our report and were previously reported [14, 21]. Although the frameshift alteration was novel, the frameshift nature of c.1508_1509insT and the predicted introduction of a termination codon of p.Thr504HisfsTer19 are predicted to be harmful. Moreover, the extracellular domains of six EC cadherin repeats were highly conserved in the amino acid sequence and shown to be critical for normal protein function. Hence, our study added further evidence to the pathology of these mutations.

In conclusion, we report three mutations in the *PCDH19* gene associated with early infantile epileptic encephalopathy. Of the three mutations, one variation was in a mosaic male and is a novel finding, the missense variation is de novo and is the second female case reported, and the nonsense variation is of paternal inheritance and is the first report in a female. Our report provides further proof and opens a new point of view for the molecular genetic diagnosis of patients with a special type of epilepsy. Perhaps more significantly is that the new mutation discovered in the mosaic male would expand the current knowledge about EIEE9 and guide diagnosis and targeted therapy in male patients. Finally, there are notable limitations to this study, including the relatively small sample size. However, this study has certainly opened a new frontier for further investigation into early infantile epileptic encephalopathy related to the *PCDH19* gene in the future.

## Additional files

Additional file 1: Detailed experimental methods for gene testing. (DOC 31 kb)

Additional file 2: The Sanger sequencing results of both strands covering the variant on Integrate Genome Viewer for patient 1.(DOC 66 kb)

## Abbreviations

AED: Antiepileptic drugs
CT: Computed tomography
DNA: Deoxyribonucleic acid
EFMR: Epilepsy and mental retardation limited to females
GTCS: Generalized tonic and clonic seizures
MRI: Brain magnetic resonance imaging
OXC: Oxcarbazepine
PB: Phenobarbital
PCDH19: Protocadherin-19
PCR: Polymerase chain reaction
SCN1A: Sodium voltage-gated channel alpha subunit 1
TPM: Topiramate
TS: Tonic seizures
VEEG: Video electroencephalogram
VPA: Valproate

## References

[1] Juberg RC, Hellman CD (1971). A new familial form of convulsive disorder and mental retardation limited to females. J Pediatr.

[2] Depienne C, Trouillard O, Bouteiller D (2011). Mutations and deletions in PCDH19 account for various familial or isolated epilepsies in females. Hum Mutat.

[3] Depienne C, LeGuern E (2012). PCDH19-related infantile epileptic encephalopathy: an unusual X-linked inheritance disorder. Hum Mutat.

[4] van Harssel JJ, Weckhuysen S, van Kempen MJ (2013). Clinical and genetic aspects of PCDH19-related epilepsy syndromes and the possible role of PCDH19 mutations in males with autism spectrum disorders. Neurogenetics.

[5] Duszyc K, Terczynska I, Hoffman-Zacharska D (2015). Epilepsy and mental retardation restricted to females: X-linked epileptic infantile encephalopathy of unusual inheritance. J Appl Genet.

[6] Depienne C, Bouteiller D, Keren B (2009). Sporadic infantile epileptic encephalopathy caused by mutations in PCDH19 resembles Dravet syndrome but mainly affects females. PLoS Genet.

[7] Marini C, Mei D, Parmeggiani L (2010). Protocadherin 19 mutations in girls with infantile-onset epilepsy. Neurology.

[8] Specchio N, Marini C, Terracciano A (2011). Spectrum of phenotypes in female patients with epilepsy due to protocadherin 19 mutations. Epilepsia.

[9] Redies C, Vanhalst K, Fv R (2005). delta-Protocadherins: unique structures and functions. Cell Mol Life Sci.

[10] Dibbens LM, Tarpey PS, Hynes K (2008). X-linked protocadherin 19 mutations cause female-limited epilepsy and cognitive impairment. Nat Genet.

[11] Hynes K, Tarpey P, Dibbens LM (2010). Epilepsy and mental retardation limited to females with PCDH19 mutations can present *de novo* or in single generation families. J Med Genet.

[12] Dibbens LM, Kneen R, Bayly MA (2011). Recurrence risk of epilepsy and mental retardation in females due to parental mosaicism of PCDH19 mutations. Neurology.

[13] Marini C, Darra F, Specchio N (2012). Focal seizures with affective symptoms are a major feature of PCDH19 gene-related epilepsy. Epilepsia.

[14] Terracciano A, Trivisano M, Cusmai R (2016). PCDH19-related epilepsy in two mosaic male patients. Epilepsia.

[15] Perez D, Hsieh DT, Rohena L (2017). Somatic mosaicism of PCDH19 in a male with early infantile epileptic encephalopathy and review of the literature. Am J Med Genet A.

[16] Thiffault I, Farrow E, Smith L (2016). PCDH19-related epileptic encephalopathy in a male mosaic for a truncating variant. Am J Med Genet A.

[17] de Lange IM, Rump P, Neuteboom RF (2017). Male patients affected by mosaic PCDH19 mutations: five new cases. Neurogenetics.

[18] Scheffer IE, Turner SJ, Dibbens LM (2008). Epilepsy and mental retardation limited to females: an under-recognized disorder. Brain.

[19] Tan C, Shard C, Ranieri E (2015). Mutations of protocadherin 19 in female epilepsy (PCDH19-FE) lead to allopregnanolone deficiency. Hum Mol Genet.

[20] Stosser MB, Lindy AS, Butler E (2018). High frequency of mosaic pathogenic variants in genes causing epilepsy-related neurodevelopmental disorders. Genet Med.

[21] Carvill GL, Heavin SB, Yendle SC (2013). Targeted resequencing in epileptic encephalopathies identifies *de novo* mutations in CHD2 and SYNGAP1. Nat Genet.

