# Supplementary material for: Chinese cases of early infantile epileptic encephalopathy: a novel mutation in the PCDH19 gene was proved in a mosaic male- case report
Source: BMC Med Genet. 2018 Jun 4;19:92. doi: 10.1186/s12881-018-0621-x (PMC5987650; doi:10.1186/s12881-018-0621-x)
Supplement: Supplementary file 1 — Detailed experimental methods for gene testing. (DOC 31 kb) [file 12881_2018_621_MOESM1_ESM.doc]

**Genomic DNA extraction**

The Qiagen FlexiGene DNA kit (Qiagen company, German) was used to extract genomic DNA from blood samples, following the guidance of manufacture. The genomic DNA extracted from blood specimen was set for storage under -20°C. The PCR reaction procedure was as follows: 95°C for 10 min, 35 cycles (95°C for 30 sec, 60°C for 30 sec, 72°C for 45 sec) followed by a final extension step at 72°C for 5 min.

**DNA library construction**

To construct the DNA library, genomic DNA sample was fragmented into 150~300bp DNA fragments by ultrasonic processor. Adaptors to both ends of these DNA fragments were ligated and cohesive ends of the DNA fragments were trimmed. The DNA library was amplified and purified by PCR.

**Hybrid capture**

The target DNA fragments from amplified DNA library were hybridized and captured by probes and then amplified through SureSelect target enrichment system (Agilgent). Then the products were purified and quantified.

**Sequencing**

Single-read sequencing was performed by NextSeq500 (illumina). Raw data were acquired in the format of Fastaq.

**Data analysis**

Raw data can be transformed into identifiable base sequence with software CASAVA (1.8.2). Then Align analysis, SNP analysis and DIP analysis were conducted to obtain information of mutation sites from targeted region. At last, protein damage analysis was conducted to qualitatively predict the probability of the results by PolyPhen-2.2.2, and thus obtaining mutation sites which need further validation.

**First-generation sequencing verification**

The gene sequences of above variation sites were acquired from GenBank. The primers were designed by the website Primer Z（http://genepipe.ncgm.sinica.edu.tw/primerz/primerz4.do）and then synthesized. The variation sites were amplified using PCR and sequenced with the first-generation sequencing. The obtained sequences were aligned with the previous results, and false positive sites obtained by NGS (the next generation sequencing) were ruled out.
