# Supplementary figures and images for: Chinese cases of early infantile epileptic encephalopathy: a novel mutation in the PCDH19 gene was proved in a mosaic male- case report
Source: BMC Med Genet. 2018 Jun 4;19:92. doi: 10.1186/s12881-018-0621-x (PMC5987650; doi:10.1186/s12881-018-0621-x)

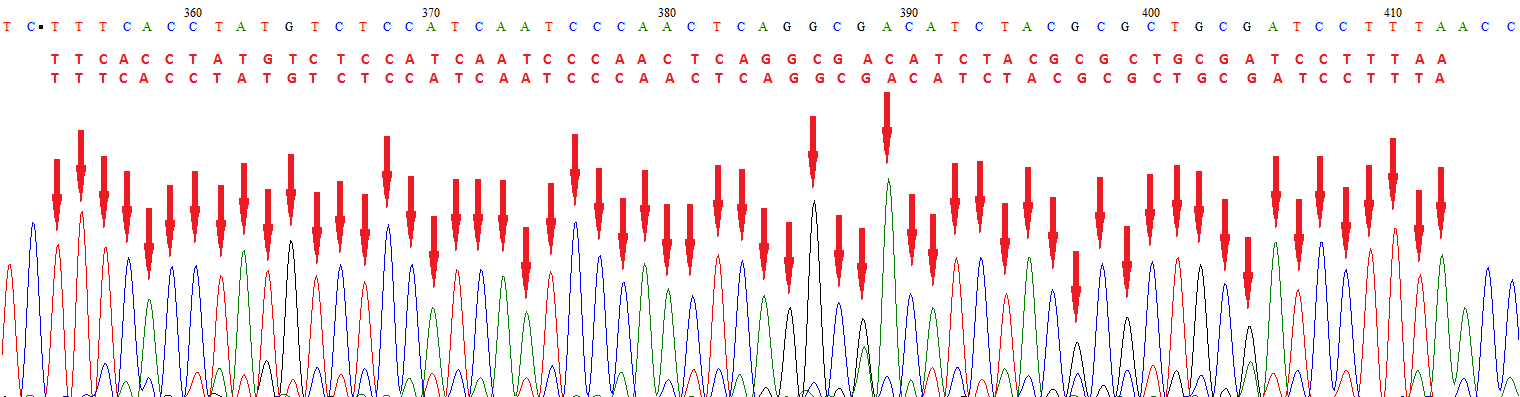


Positive genome sequence of patient 1


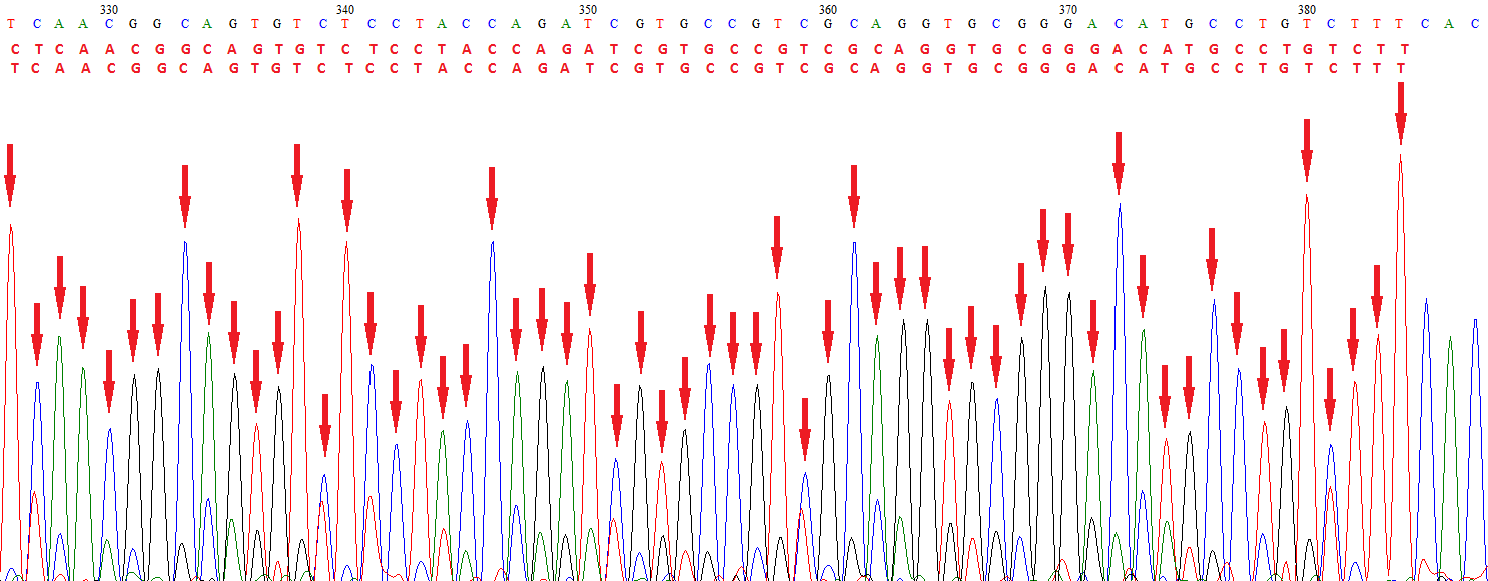


Reverse genome sequence of patient 1

Supplement: Supplementary file 2 — The Sanger sequencing results of both strands covering the variant on Integrate Genome Viewer for patient 1.(DOC 66 kb) [file 12881_2018_621_MOESM2_ESM.doc]
